# Molecular Typing of *Treponema pallidum*: A Systematic Review and Meta-Analysis

**DOI:** 10.1371/journal.pntd.0001273

**Published:** 2011-11-08

**Authors:** Rui-Rui Peng, Alberta L. Wang, Jing Li, Joseph D. Tucker, Yue-Ping Yin, Xiang-Sheng Chen

**Affiliations:** 1 National Center for STD Control, Chinese Academy of Medical Sciences and Peking Union Medical College Institute of Dermatology, Nanjing, China; 2 The University of Texas Health Science Center at Houston, Houston, Texas, United States of America; 3 Infectious Disease Unit, Massachusetts General Hospital, Boston, Massachusetts, United States of America; University of Washington, United States of America

## Abstract

**Background:**

Syphilis is resurgent in many regions of the world. Molecular typing is a robust tool for investigating strain diversity and epidemiology. This study aimed to review original research on molecular typing of *Treponema pallidum* (*T. pallidum*) with three objectives: (1) to determine specimen types most suitable for molecular typing; (2) to determine *T. pallidum* subtype distribution across geographic areas; and (3) to summarize available information on subtypes associated with neurosyphilis and macrolide resistance.

**Methodology/Principal Findings:**

Two researchers independently searched five databases from 1998 through 2010, assessed for eligibility and study quality, and extracted data. Search terms included “*Treponema pallidum*,” or “syphilis,” combined with the subject headings “molecular,” “subtyping,” “typing,” “genotype,” and “epidemiology.” Sixteen eligible studies were included. Publication bias was not statistically significant by the Begg rank correlation test. Medians, inter-quartile ranges, and 95% confidence intervals were determined for DNA extraction and full typing efficiency. A random-effects model was used to perform subgroup analyses to reduce obvious between-study heterogeneity. Primary and secondary lesions and ear lobe blood specimens had an average higher yield of *T. pallidum* DNA (83.0% *vs.* 28.2%, χ^2^ = 247.6, *p*<0.001) and an average higher efficiency of full molecular typing (80.9% *vs.* 43.1%, χ^2^ = 102.3, *p*<0.001) compared to plasma, whole blood, and cerebrospinal fluid. A pooled analysis of subtype distribution based on country location showed that 14d was the most common subtype, and subtype distribution varied across geographic areas. Subtype data associated with macrolide resistance and neurosyphilis were limited.

**Conclusions/Significance:**

Primary lesion was a better specimen for obtaining *T. pallidum* DNA than blood. There was wide geographic variation in *T. pallidum* subtypes. More research is needed on the relationship between clinical presentation and subtype, and further validation of ear lobe blood for obtaining *T. pallidum* DNA would be useful for future molecular studies of syphilis.

## Author Summary

Syphilis has been resurgent in many parts of the world in past decades. Understanding the epidemiology of syphilis is important for estimating disease burdens, monitoring epidemic trends, and evaluating intervention activities. *Treponema pallidum* (*T. pallidum*), the pathogen of syphilis, cannot be grown *in vitro*. Because *T. pallidum* cannot be cultured, molecular typing of *T. pallidum* is particularly useful and allows for investigation of infection diversity and epidemiology. We conducted a statistical analysis of available published data to investigate the current research progress of molecular typing of syphilis. Our analysis showed that primary lesion was a better specimen for obtaining *T. pallidum* DNA than blood. Blood specimens collected from scraping the ear lobes had high yield of *T. pallidum* DNA and high full typing efficiency. Ear lobe blood is a promising specimen for future *T. pallidum* molecular typing, but further research should verify this finding using a larger sample size. Within all studies, subtype 14d was most prevalent, and subtype distribution varied across geographic areas. Subtype data associated with macrolide resistance and neurosyphilis were limited. More research on molecular typing of *T. pallidum* can be useful for investigating syphilis epidemiology and designing syphilis control strategies.

## Introduction

Syphilis has been resurgent in many parts of the world in past decades [1]–[3]. This important sexually transmitted infection can facilitate the transmission of HIV infection [4], [5], increase the risk of adverse pregnancy outcomes [6], and cause substantial economic impact [7], [8]. Understanding the epidemiology of syphilis is important for estimating disease burdens, monitoring epidemic trends, and evaluating intervention activities.

Molecular typing is a powerful tool for determining diversity and epidemiology of infections, especially for *Treponema pallidum* (*T. pallidum*), an organism that cannot be cultured *in vitro* [9]. In addition, molecular typing has the potential to enhance clinical care, prevention, and control efforts by contributing to a better understanding of *T. pallidum* acquisition and transmission [10]. The first molecular typing method was introduced by the United States Centers for Disease Control and Prevention (U.S. CDC) and is based on the interstrain variability of acidic repeat protein gene (*arp*) and *T. pallidum* repeat gene subfamily II (*tprE*, *G* and *J*, hereinafter referred to as *tpr*) [11]. The typing result is named subtype [11]. Besides the above two genes, a recent study in San Francisco introduced a third gene named *rpsA* that could be targeted to improve the discriminatory ability of the typing system or to further delineate the common strain type [12]. Moreover, another recent study developed a third gene named *tp0548* with a better discriminatory typing power, and the typing result is named strain type [13].

Previous studies of *T. pallidum* molecular typing have used multiple specimens from patients with different stages of syphilis. It has been reported that specimens from moist skin lesions have a higher yield of typeable DNA [14], [15], that the lower efficiency of *arp* gene PCR assay may be related to poor full typing efficiency [14], [16], and that specific *T. pallidum* subtypes are likely associated with macrolide resistance or neurosyphilis [12], [13], [17]–[19]. This study aimed to systematically review and investigate the published research on molecular typing of *T. pallidum* in order to: (1) determine more suitable specimen types for the molecular epidemiological study of syphilis; (2) determine *T. pallidum* subtype distribution across geographic areas; and (3) summarize available information on subtypes associated with neurosyphilis and macrolide resistance.

## Methods

### Literature search

Two independent researchers (RRP and JL) searched five databases (PubMed, Embase, EBSCO, Google Scholar, and CNKI) to identify published studies from 1998, when the first typing method was introduced, through 2010. Search terms included “*Treponema pallidum*,” or “syphilis,” combined with the subject headings “molecular,” “subtyping,” “typing,” “genotype,” and “epidemiology.” References cited in the retrieved articles were evaluated for inclusion, but duplicate reports were excluded. The search was conducted in four stages (identification, screening, eligibility, and inclusion) according to PRISMA guidelines [20], [21].

### Eligibility criteria and validity assessment

The inclusion criteria consisted of the following items: (1) original studies published from 1998 through 2010 in any language; (2) description of the source of clinical specimens; (3) utilization of the *arp* and *tpr* genes, or an additional third gene for molecular typing; (4) description of typing methods; and (5) report of absolute number of each subtype category. Two researchers (RRP and JL) assessed the eligibility and validity of the studies independently according to the criteria. Any disagreement was resolved by involving of the third researcher (ALW).

### Data extraction

We extracted the following data from each study using a standardized form (Table 1): (1) first author and publication year; (2) country and location where the study was conducted; (3) study population; (4) specimen collection period; (5) clinical stage of syphilis; (6) specimen type (primary ulcer, secondary lesion, whole blood, plasma, blood collected from scraping the ear lobe [hereinafter referred to as ear lobe scraping], and cerebrospinal fluid [CSF]); (7) gene for confirming *T. pallidum* DNA in PCR assay (*tpp47*, *bmp* or *polA*); (8) number of specimens collected, and number of each type of specimen collected, if available; (9) number of specimens with positive *T. pallidum* DNA, and number of each type of specimen with positive *T. pallidum* DNA, if available; (10) number of specimens with positive amplification of *arp* or *tpr*; (11) number of fully-typed specimens, and number of each type of fully-typed specimen, if available (fully-typed specimen is specimen that can be fully typed by two genes–*arp* and *tpr* or by three genes–*arp*, *tpr*, *and rpsA* or *tp0548*); (12) number of each subtype identified; (13) macrolide resistance data, if available; and (14) subtype associated with neurosyphilis, if available.

**Table 1:** Overview of 16 studies on molecular typing of *T. pallidum* clinical strains.

First author, publication year	Country, location, study population^a^	Specimen collection period	Clinical stage of syphilis^b^	Specimen type^c^	Gene for *T. pallidum* detection^d^	No. of specimens					No. of subtypes identified
						All	DNA +	*arp *+	*tpr *+	Full type^e^	
Pillay A, 1998 [11] f	U.S., 10 cities, GUD patients; Madagascar, primary syphilis; South Africa, 3 cities, GUD patients	N/A	P	PU	*tpp47*	N/A	55	55	38	38	7; 8; 3
Sutton MY, 2001 [24]	U.S., Arizona, SP	03/1998–10/1999	P, S, L	PU, WB	*polA*	85	56	N/A	N/A	45	10
Pope V, 2005 [25]	U.S., North and South Carolina, SP	11/1999–01/2003	P, S	PU, SL	*polA*	61	27	N/A	N/A	23	7
Katz KA, 2010 [12] g	U.S., San Francisco, SP	11/2004–11/2007	P, S	PU, SL	*polA*	74	71	69	70	69	8
Marra CM, 2010 [13] h	U.S., Seattle, 87% MSM; Madagascar; U.S., San Francisco; U.S., Baltimore; China, Nanjing; Ireland, Dublin	1999–2008; 2003–2008; 2001–2007; 1999–2001; 2006–2007; 2002	P, S, L	PU, SL, WB, CSF	N/A	N/A	N/A	N/A	N/A	84; 20; 19; 15; 10; 10	8; 6; 4; 5; 2; 4
Martin IE, 2010 [18]	Canada, Alberta and Northwest territories, SP	02/2007–04/2009	P, S, C	PU, SL, WB, PS^i^, SS^i^, CSF^i^, VEF^i^	*bmp, tpp47* and *polA*	449	43	43	36	36	4
Cruz AR, 2010 [26]	Colombia, Cali, from a network of public sector primary health care providers	2003–2009	S	SL, WB	*polA*	38	20	6	8	6	4
Zeng TB, 2004 [27]	China, Hengyang and Jiangmen, SP	02/2002–01/2004	P	PU	*polA*	85	69	57	63	57	8
Zhan LS, 2005 [28]	China, South Hunan Province, SP	02/2002–08/2004	P	PU	*polA*	52	43	43	41	38	10
Zheng HP, 2005 [29]	China, Guangzhou, MSP	2002–2004	P	PU	*bmp*	62	54	47	49	47	7
Martin IE, 2009 [17]	China, Shanghai, GUD patients	12/2007–05/2008	P	PU, WB^i^	*bmp, tpp47* and *polA*	57	38	36	38	36	4
Pillay A, 2002 [16]	South Africa, 5 cities, MSP	1996–2000	P	PU	*tpp47* or *polA*	1954	201	161	175	161	35
Molepo J, 2007 [19]	South Africa, Pretoria, patients in neurology ward	06/1999–09/2000	LN	CSF	*tpp47*	50	28	13	15	13	4
Florindo C, 2008 [14]	Portugal, Lisbon, SP	2004–2007	P, S	PU, SL, WB	*bmp* and *polA*	N/A	86	N/A	N/A	42	3
Castro R, 2009 [15]	Portugal, Lisbon, SP	06/2003–07/2005	P, S, L	PU, SL, WB, PS, ELS	*polA*	212	90	N/A	N/A	62	5
Cole MJ, 2009 [30]	U.K., Scotland, MSM	08/2006–12/2007	P, S	GU, AU, OU	*polA*	N/A	75	61	64	58	6

a Study population: GUD-genital ulcer disease, SP-STD patients, including males and females, MSP-male STD patients, and MSM-men who have sex with men.

b Clinical stage of syphilis: P-primary syphilis, S-secondary syphilis, L-latent syphilis, C-congenital syphilis, and LN-late neurosyphilis.

c Specimen type: PU-primary ulcer, WB-whole blood, SL-secondary lesion, including secondary skin lesion and/or mucosal lesion, CSF-cerebrospinal fluid, PS-plasma specimen, SS-serum specimen, VEF-vitreous eye fluid, ELS-ear lobe scraping, GU-genital ulcer, AU-anal ulcer, and OU-oral ulcer.

d Gene for *T. pallidum* detection: *tpp47*-47 kDa protein gene, *bmp*-basic membrane protein gene, and *polA*-DNA polymerase I gene.

e Full type was based on two genes (*arp* and *tpr*) or three genes (*arp*, *tpr*, and *rpsA or tp0548*).

f Eight laboratory strains were excluded, remaining 55 clinical strains were included for analysis.

g Introducing a third gene, *rpsA*.

h Introducing a third gene, *tp0548*. Laboratory strains were excluded.

i *T. pallidum* DNA was not amplified successfully by screening PCR assay.

### Statistical analysis

DNA extraction efficiency was defined as a proportion of *T. pallidum* positive specimens out of all extracted specimens. Molecular typing efficiency was defined as a proportion of fully-typed specimens out of *T. pallidum* positive specimens. We performed a pooled analysis of subtype distribution by country location. One study identified subtypes in three countries (U.S., Madagascar, and South Africa), so the subtypes were disaggregated [11].

We used Statistical Package for the Social Sciences for Windows (SPSS, version 18.0, Chicago, IL, USA) and Comprehensive Meta-Analysis software (CMA, version 2.0, Biostat Inc., Englewod, NJ, USA) for statistical analysis. Point estimates with corresponding 95% confidence intervals (CI) for DNA extraction efficiency and typing efficiency were carried out for each individual study if available. A chi-square test (*p*<0.05 indicating statistical significance) was applied to compare the different categories. Q test (*p*<0.10 indicating statistical significance) and I^2^ value (ranging between 0% and 100%, with lower value representing less heterogeneity) were calculated to measure between-study heterogeneity [22]. A random-effects model was used to perform the subgroup analysis. Publication bias was assessed by the Begg rank correlation test (*p*<0.05 indicating statistical significance) [23].

## Results

### Study selection

**Figure 1:**
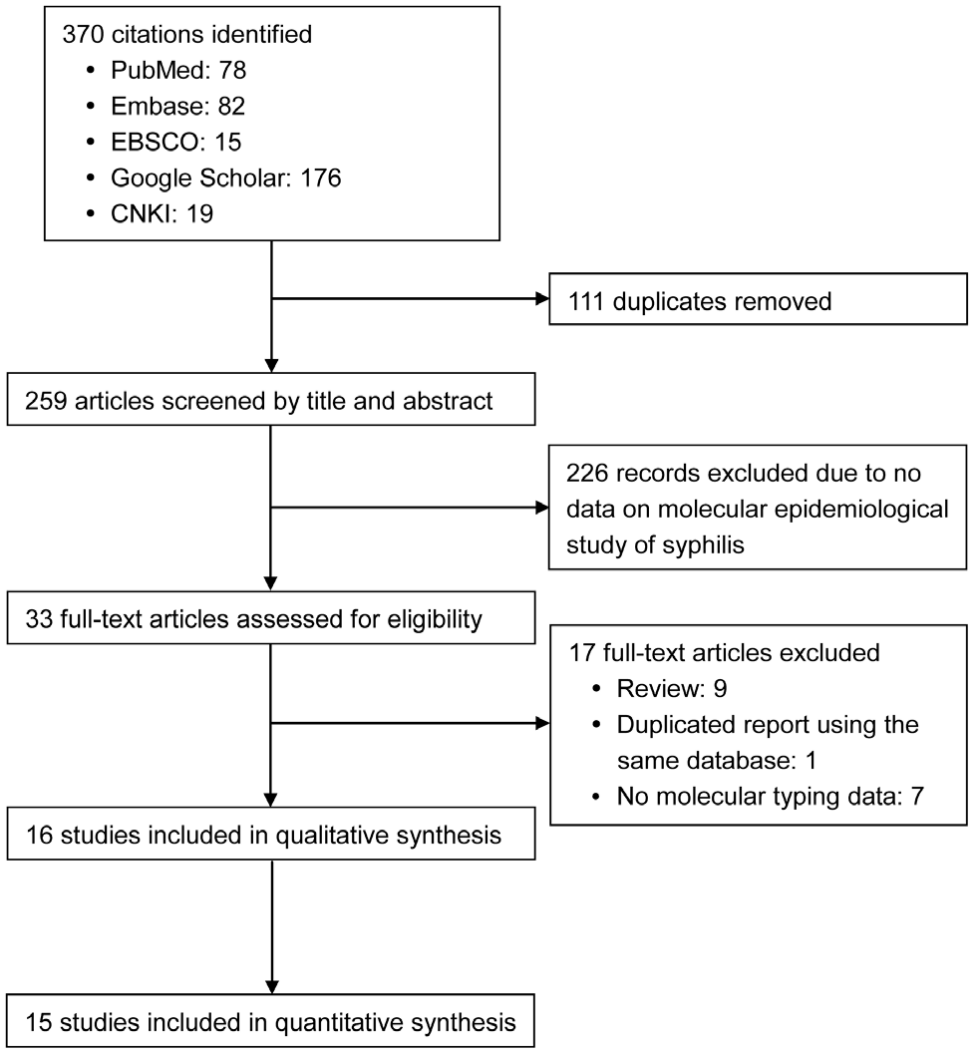
Search strategy of published studies according to PRISMA guidelines.

As shown in Figure 1, 370 potential abstracts were identified, and 111 duplicate records were removed. Of the remaining abstracts, all were screened, and 226 that did not study the molecular typing of syphilis were excluded. Thirty-three full-text articles were assessed for eligibility and of those, 16 studies were included (Table 1) [11]–[19], [24]–[30]. No additional eligible studies were identified by checking the references of retrieved articles. Fourteen studies used two genes (*arp* and *tpr*) for molecular typing [11], [14]–[19], [24]–[30], and two recent studies used three genes (*arp*, *tpr*, and *rpsA* or *tp0548*) [12], [13].

### DNA extraction efficiency

**Figure 2:**
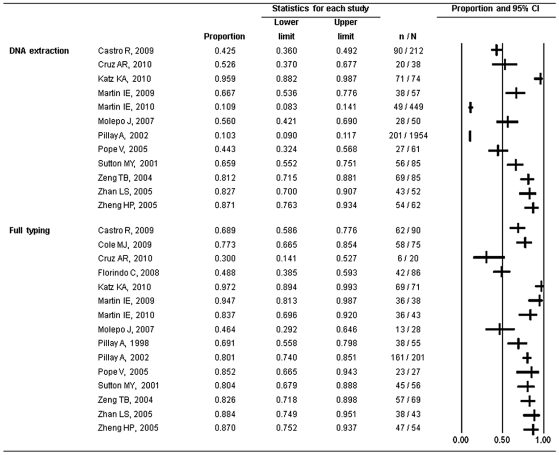
Forest plot of DNA extraction and molecular typing efficiency from 15 studies. Proportion represents DNA extraction efficiency or full typing efficiency. Lower limit and upper limit represent 95% confidence intervals.

DNA extraction efficiency ranged from 10.3% to 95.9% based on 12 studies (Figure 2) [12], [15]–[19], [24]–[29]. The median was 60.9% with an inter-quartile range (IQR) of 43.0%–82.3%. Blood specimens resulted in a lower yield of *T. pallidum* DNA compared to skin specimens (30.0% *vs.* 85.7%, χ^2^ = 245.2, *p*<0.001). No obvious publication bias was observed (Begg rank correlation test, *p* = 0.11).

Strong evidence of heterogeneity (I^2^ = 98.4%, *p*<0.001) was observed between studies. Subgroup analysis by specimen type partly reduced the heterogeneity (Table 2). Primary and secondary lesions and ear lobe blood specimens had an average higher yield of *T. pallidum* DNA (83.0% *vs.* 28.2%, χ^2^ = 247.6, *p*<0.001) compared to plasma, whole blood and CSF. DNA extraction from CSF was more efficient than from whole blood and plasma (33.6% *vs.* 24.5%, χ^2^ = 13.4, *p*<0.001). Whole blood and plasma had the lowest DNA extraction efficiency, with no significant difference between the two (25.0% *vs.* 13.0%, χ^2^ = 1.0, *p* = 0.32).

**Table 2:** Subgroup analysis of DNA extraction and molecular typing efficiency by specimen type.

Specimen type	Efficiency % (95% CI)	No. of studies	Heterogeneity	
			I^2^ (%)	*p*-value
**DNA extraction**				
Primary ulcer	86.4 (80.0–90.9)	7	48.9	0.07
Secondary lesion	75.0 (57.8–86.8)	4	0	0.71
Ear lobe scraping^a^	65.6 (47.9–79.8)	1		
Plasma	13.0 (0.5–81.2)	2	82.8	0.02
Whole blood	25.0 (13.5–41.6)	5	76.7	0.002
Cerebrospinal fluid	33.6 (4.1–85.6)	2	67.5	0.08
**Molecular typing**				
Primary ulcer	82.8 (75.3–88.3)	9	66.7	0.002
Secondary lesion	71.9 (50.2–86.6)	4	0	0.57
Ear lobe scraping^a^	76.2 (54.0–89.7)	1		
Plasma	62.5 (44.9–77.3)	1		
Whole blood	34.5 (17.7–56.4)	4	65.0	0.04
Cerebrospinal fluid	46.4 (29.2–64.6)	1		

a Blood collected from scraping the ear lobe.

When the blood specimens were disaggregated by clinical stage based on three studies, blood specimens from patients with secondary syphilis had higher yield of DNA than blood from patients with primary or latent syphilis (55.8% *vs.* 34.1% *vs.* 33.6%, χ^2^ = 7.3, *p* = 0.007) [15], [17], [26].

### Molecular typing efficiency

The difference of PCR efficiency between the *arp* and *tpr* genes was not statistically significant based on 11 studies (χ^2^ = 5.2, *p* = 0.88) [11], [12], [16]–[19], [26]–[30]. Typing efficiency ranged from 30.0% to 97.2% among 15 studies (Figure 2) [11], [12], [14]–[19], [24]–[30], with the median of 80.4% and IQR of 68.9%–87.0%. Publication bias was not statistically significant (Begg rank correlation test, *p* = 0.11).

Subgroup analysis by specimen type was also conducted to reduce the obvious heterogeneity between studies (I^2^ = 84.7%, *p*<0.001) (Table 2). Primary and secondary lesions and ear lobe blood specimens had an average higher efficiency of full molecular typing (80.9% *vs.* 43.1%, χ^2^ = 102.3, *p*<0.001) compared to plasma, whole blood, and CSF. Plasma ranked in the middle of all blood specimens in terms of typing efficiency. The typing efficiency of whole blood was the lowest, with no significant difference compared with CSF (34.5% *vs.* 46.4%, χ^2^ = 1.3, *p* = 0.25).

One study that disaggregated specimens by clinical stage showed that molecular typing efficiency was borderline insignificance between specimens from primary, secondary, and latent syphilis (85.7% *vs.* 83.3% *vs.* 55.1%, χ^2^ = 6.2, *p* = 0.05) [15].

### Subtype distribution

**Figure 3:**
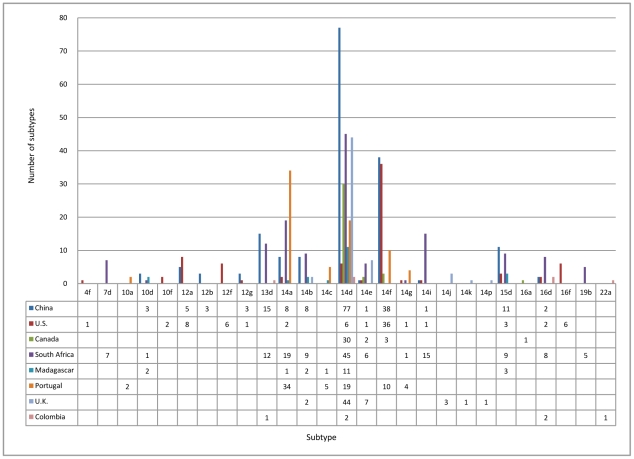
Distribution of the most common subtypes across eight geographic areas from 14 studies.

Fifty-seven subtypes of *T. pallidum* were identified from 14 studies [11], [14]–[19], [24]–[30]. For the *arp* gene, a range of 2 to 22 tandem repeats (except 9 and 21) were found. For the *tpr* genes, patterns a to m and p were found. Additionally, for the *tp0548* gene, sequences c to g and i were found [13]. For the *rpsA* gene, a range of 8 to 10 and 12 tandem repeats were found [12]. South Africa, the U.S., and China had the most abundant variety of subtypes, and 38 subtypes were identified in 177 specimens, 19 subtypes were identified in 81 specimens, and 15 subtypes were identified in 178 specimens, respectively. The pooled analysis based on country showed that the distribution of the 27 most common subtypes had substantial geographic variation (Figure 3). Overall, 14d, 14f, 14a, 13d, and 15d were most prevalent. The limited data on subtypes associated with neurosyphilis and macrolide resistance precluded completion of one study aimed to investigate the neuroinvasive and macrolide resistant subtypes.

## Discussion

The World Health Organization (WHO) recently estimated 10.6 million new cases of syphilis each year, and the emergence of macrolide resistant strains has increased the importance of molecular epidemiological investigations [31], [32]. Globally, molecular typing of *T. pallidum* clinical strains has helped characterize syphilis outbreaks [24], [30], evaluate subtypes associated with neurosyphilis [13], [19], monitor macrolide resistance [12], [17], [18], differentiate between relapse and re-infection episodes [13], and better understand the geographic, temporal, and population distributions of *T. pallidum* [11], [13], [30]. Despite the public health and clinical benefits of molecular investigation of syphilis, limited numbers of studies in a few epidemic countries have focused on the molecular typing of *T. pallidum* since the first typing assay appeared.

Our review showed that extracting DNA from blood specimens resulted in a lower yield compared to skin lesions. This is consistent with another study that directly compared the two methods [33]. Previous studies indicated that this may be largely related to the lower *T. pallidum* load in blood than that in skin lesions [9], [34]. Moreover, PCR-inhibitory substances are more likely to exist in whole blood [35]. Our analysis showed that moist skin lesions from patients with primary or secondary syphilis were suitable for molecular investigation of syphilis. Additionally, ear lobe blood specimen could be an alternative when there are no visible skin lesions.

Previous studies reported results of partial molecular typing due to low success rate of the *arp* gene PCR assay [14], [16], [36]. Our analysis revealed that the efficiency of PCR assay between the *arp* and *tpr* genes was not statistically significant. The specimens that had most efficient molecular typing were the same specimens that yielded higher *T. pallidum* DNA–primary ulcer, secondary lesion, and ear lobe scraping. CSF from patients with late neurosyphilis resulted in 46.4% typing efficiency. Although the typing efficiency is not high, the typing results of CSF highlight the potential for typing neuroinvasive strains. Interestingly, ear lobe scrapings had the highest DNA yield and typing efficiency among blood specimens, with no significant difference compared with primary ulcers and secondary lesions. Because the ear lobe is rich in capillaries, poor in sensory nerves, and can be easily accessed [37], it has promising prospect for blood specimen collection. Since there has been only one study verifying the molecular typing efficiency of ear lobe blood specimens, the results should be validated using a larger sample size.

A surprising level of genetic diversity of *T. pallidum* was evident, with predominance of several subtypes worldwide. 14d was most prevalent, except in the U.S. (ranked third) and Portugal (ranked second). The abundant variety in subtype distribution across geographic areas could reflect regional sexual network patterns. However, the predominance of 14d may indicate some linked transmission, and 14d may be an original circulating subtype in many parts of the world.

The association between specific subtypes and neurosyphilis can lead to a detailed understanding of the molecular mechanisms underlying neurosyphilis, and neuroinvasive subtypes can be a laboratory marker for increased risk of neurosyphilis. Though successful typing from CSF has made this kind of research possible, data is still limited. Our systematic literature search identified only two studies on CSF typing. One identified 14a, 3e, 2i, and 17e in CSF from patients with late neurosyphilis [19]. Another study showed that 14d/f was significantly associated with neurosyphilis when compared with other strain types (*p* = 0.02) [13]. However, the typing efficiency of CSF specimens was relatively lower than other specimen types, and the characteristics of specimens in which subtypes could not be identified were not available. Future investigations using a larger sample size and more sensitive typing method for CSF are warranted.

A single mutation conferring macrolide resistance of *T. pallidum* has been reported in the U.S. [12], [38]–[40], Dublin [38], Canada [18], [33], [41], Shanghai [17], [42], and the Czech Republic [43], [44]. However, resistance has not been found in some African countries (Madagascar, Tanzania, and Uganda) [45]–[47]. Previous studies showed that antibiotic selection may contribute to increased macrolide resistance [39], [40], and resistant mutations were present in at least 2 separate strains of *T. pallidum* using a molecular marker (51 base pair insertion) [39]. Further investigation of resistant subtypes using molecular typing can help elucidate the molecular mechanism of macrolide resistance, but data is still not abundant. Three of the included studies mentioned resistant subtypes. One study in Shanghai found 100% (38 patients) macrolide resistance, and subtype 14f was predominant [17]. Resistance rate was 19.4% (7/36) in West Canada, and all resistant subtypes were 14d [18]. In San Francisco, 67.7% (42/62) were macrolide resistance, and subtype 14d9 was predominant [12].

To our knowledge, this is the first literature review and meta-analysis of globally published papers on molecular typing of *T. pallidum*. Because the quality of included studies varied, the following limitations should be acknowledged. First, the sample size of fully-typed specimens was small in most studies (median of 44 and IQR of 36–61), resulting in limited statistical power and limited information on transmission networks. Second, although stratified analysis can partly reduce the between-study heterogeneity, modest heterogeneity still existed. This may have been due to study-specific factors, such as specimen quality and laboratory condition. Third, because genital specimens were available more easily from males than females, the enrollment of males was predominant in the included studies, which used genital ulcers for typing. Differences in subtype distribution between males and females may have not been detected. Finally, our study included only published studies and abstracted data from articles, not raw data, which may have resulted in some selection bias.

Future molecular epidemiological research of syphilis should be informative for effective syphilis prevention and control programs. Possible studies should be at least focused on: (1) identification of high-risk populations to trace transmission networks and treat high-risk infection sources; (2) verification of subtypes associated with macrolide resistance and neurosyphilis to aid diagnosis and treatment; and (3) research on the invasiveness and virulence of different *T. pallidum* subtypes in order to better understand of the pathology of syphilis.

## Supporting Information

Checklist S1 **PRISMA Checklist.**
(DOC)

## References

[1] Fenton KA, Breban R, Vardavas R, Okano JT, Martin T (2008). Infectious syphilis in high-income settings in the 21st century.. Lancet Infect Dis.

[2] Chen ZQ, Zhang GC, Gong XD, Lin C, Gao X (2007). Syphilis in China: results of a national surveillance programme.. Lancet.

[3] Tucker JD, Chen XS, Peeling RW (2010). Syphilis and social upheaval in China.. N Engl J Med.

[4] Buchacz K, Patel P, Taylor M, Kerndt PR, Byers RH (2004). Syphilis increases HIV viral load and decreases CD4 cell counts in HIV-infected patients with new syphilis infections.. AIDS.

[5] Zetola NM, Klausner JD (2007). Syphilis and HIV infection: an update.. Clin Infect Dis.

[6] Doroshenko A, Sherrard J, Pollard AJ (2006). Syphilis in pregnancy and the neonatal period.. Int J STD AIDS.

[7] Pultorak E, Wong W, Rabins C, Mehta SD (2009). Economic burden of sexually transmitted infections: incidence and direct medical cost of Chlamydia, gonorrhea, and syphilis among Illinois adolescents and young adults, 2005–2006.. Sex Transm Dis.

[8] Schmid G (2004). Economic and programmatic aspects of congenital syphilis prevention.. Bull World Health Organ.

[9] Lafond RE, Lukehart SA (2006). Biological basis for syphilis.. Clin Microbiol Rev.

[10] Morshed MG, Lee MK, Jorgensen D, Isaac-Renton JL (2007). Molecular methods used in clinical laboratory: prospects and pitfalls.. FEMS Immunol Med Microbiol.

[11] Pillay A, Liu H, Chen CY, Holloway B, Sturm AW (1998). Molecular subtyping of *Treponema pallidum* subspecies *pallidum*.. Sex Transm Dis.

[12] Katz KA, Pillay A, Ahrens K, Kohn RP, Hermanstyne K (2010). Molecular epidemiology of syphilis-San Francisco, 2004–2007.. Sex Transm Dis.

[13] Marra CM, Sahi SK, Tantalo LC, Godornes C, Reid T (2010). Enhanced molecular typing of *Treponema pallidum*: geographical distribution of strain types and association with neurosyphilis.. J Infect Dis.

[14] Florindo C, Reigado V, Gomes JP, Azevedo J, Santo I (2008). Molecular typing of *Treponema pallidum* clinical strains from Lisbon, Portugal.. J Clin Microbiol.

[15] Castro R, Prieto E, Aguas MJ, Manata MJ, Botas J (2009). Molecular subtyping of *Treponema pallidum* subsp. *pallidum* in Lisbon, Portugal.. J Clin Microbiol.

[16] Pillay A, Liu H, Ebrahim S, Chen CY, Lai W (2002). Molecular typing of *Treponema pallidum* in South Africa: cross-sectional studies.. J Clin Microbiol.

[17] Martin IE, Gu W, Yang Y, Tsang RS (2009). Macrolide resistance and molecular types of *Treponema pallidum* causing primary syphilis in Shanghai, China.. Clin Infect Dis.

[18] Martin IE, Tsang RS, Sutherland K, Anderson B, Read R (2010). Molecular typing of *Treponema pallidum* strains in western Canada: predominance of 14d subtypes.. Sex Transm Dis.

[19] Molepo J, Pillay A, Weber B, Morse SA, Hoosen AA (2007). Molecular typing of *Treponema pallidum* strains from patients with neurosyphilis in Pretoria, South Africa.. Sex Transm Infect.

[20] Moher D, Liberati A, Tetzlaff J, Altman DG (2009). Preferred reporting items for systematic reviews and meta-analyses: the PRISMA statement.. PLoS Med.

[21] Liberati A, Altman DG, Tetzlaff J, Mulrow C, Gotzsche PC (2009). The PRISMA statement for reporting systematic reviews and meta-analyses of studies that evaluate health care interventions: explanation and elaboration.. PLoS Med.

[22] Higgins JP, Thompson SG, Deeks JJ, Altman DG (2003). Measuring inconsistency in meta-analyses.. BMJ.

[23] Borenstein M, Hedges LV, Higgins JP, Rothstein HR, Borenstein M, Hedges LV, Higgins JP, Rothstein HR (2009). Publication bias.. Introduction to Meta-analysis.

[24] Sutton MY, Liu H, Steiner B, Pillay A, Mickey T (2001). Molecular subtyping of *Treponema pallidum* in an Arizona County with increasing syphilis morbidity: use of specimens from ulcers and blood.. J Infect Dis.

[25] Pope V, Fox K, Liu H, Marfin AA, Leone P (2005). Molecular subtyping of *Treponema pallidum* from North and South Carolina.. J Clin Microbiol.

[26] Cruz AR, Pillay A, Zuluaga AV, Ramirez LG, Duque JE (2010). Secondary syphilis in Cali, Colombia: new concepts in disease pathogenesis.. PLoS Negl Trop Dis.

[27] Zeng TB, Wu YM, Huang SJ, Wu ZZ (2004). Preliminary study on molecular subtyping of *Treponema pallidum* in Hengyang and Jiangmen regions.. Chin J Dermatol.

[28] Zhan LS, Zeng TB, Yan JL, Yang TS, Wu SY (2005). Preliminary study on molecular subtyping of *Treponema pallidum* in south Hunan area.. Practical Preventive Medicine.

[29] Zheng HP, Ou ZY, Hu YS, Huang JM, Li ML (2005). Detection and genotyping of *Treponema pallidum* by a nested PCR.. Chin J Dermatol.

[30] Cole MJ, Chisholm SA, Palmer HM, Wallace LA, Ison CA (2009). Molecular epidemiology of syphilis in Scotland.. Sex Transm Infect.

[31] Schmid G, Rowley JT, Samuelson J, Tun Y, Guraiib M (2009). World Health Organization (WHO) 2005 Global Estimates of the Incidence and Prevalence of Sexually Transmitted Infections (STIs)..

[32] Stamm LV (2010). Global challenge of antibiotic-resistant *Treponema pallidum*.. Antimicrob Agents Chemother.

[33] Martin IE, Tsang RS, Sutherland K, Tilley P, Read R (2009). Molecular characterization of syphilis in patients in Canada: azithromycin resistance and detection of *Treponema pallidum* DNA in whole-blood samples versus ulcerative swabs.. J Clin Microbiol.

[34] Salazar JC, Rathi A, Michael NL, Radolf JD, Jagodzinski LL (2007). Assessment of the kinetics of *Treponema pallidum* dissemination into blood and tissues in experimental syphilis by real-time quantitative PCR.. Infect Immun.

[35] Al-Soud WA, Radstrom P (2001). Purification and characterization of PCR-inhibitory components in blood cells.. J Clin Microbiol.

[36] Liu H, Rodes B, George R, Steiner B (2007). Molecular characterization and analysis of a gene encoding the acidic repeat protein (*Arp*) of *Treponema pallidum*.. J Med Microbiol.

[37] Castro R, Prieto E, Aguas MJ, Manata MJ, Botas J (2007). Detection of *Treponema pallidum* sp *pallidum* DNA in latent syphilis.. Int J STD AIDS.

[38] Lukehart SA, Godornes C, Molini BJ, Sonnett P, Hopkins S (2004). Macrolide resistance in *Treponema pallidum* in the United States and Ireland.. N Engl J Med.

[39] Marra CM, Colina AP, Godornes C, Tantalo LC, Puray M (2006). Antibiotic selection may contribute to increases in macrolide-resistant *Treponema pallidum*.. J Infect Dis.

[40] Mitchell SJ, Engelman J, Kent CK, Lukehart SA, Godornes C (2006). Azithromycin-resistant syphilis infection: San Francisco, California, 2000–2004.. Clin Infect Dis.

[41] Morshed MG, Jones HD (2006). *Treponema pallidum* macrolide resistance in BC.. CMAJ.

[42] Zhou P, Li K, Lu H, Qian Y, Gu X (2010). Azithromycin treatment failure among primary and secondary syphilis patients in Shanghai.. Sex Transm Dis.

[43] Woznicova V, Matejkova P, Flasarova M, Zakoucka H, Valisova Z (2010). Clarithromycin treatment failure due to macrolide resistance in *Treponema pallidum* in a patient with primary syphilis.. Acta Derm Venereol.

[44] Matejkova P, Flasarova M, Zakoucka H, Borek M, Kremenova S (2009). Macrolide treatment failure in a case of secondary syphilis: a novel A2059G mutation in the 23S rRNA gene of *Treponema pallidum* subsp. *pallidum*.. J Med Microbiol.

[45] Van Damme K, Behets F, Ravelomanana N, Godornes C, Khan M (2009). Evaluation of azithromycin resistance in *Treponema pallidum* specimens from Madagascar.. Sex Transm Dis.

[46] Riedner G, Rusizoka M, Todd J, Maboko L, Hoelscher M (2005). Single-dose azithromycin versus penicillin G benzathine for the treatment of early syphilis.. N Engl J Med.

[47] Kiddugavu MG, Kiwanuka N, Wawer MJ, Serwadda D, Sewankambo NK (2005). Effectiveness of syphilis treatment using azithromycin and/or benzathine penicillin in Rakai, Uganda.. Sex Transm Dis.

